# A scientometric study of the research on ion exchange membranes†

**DOI:** 10.1039/c8ra04686g

**Published:** 2018-07-02

**Authors:** Shanxue Jiang, Kimberly F. L. Hagesteijn, Jin Ni, Bradley P. Ladewig

**Affiliations:** Barrer Centre, Department of Chemical Engineering, Imperial College London UK b.ladewig@imperial.ac.uk; School of Chemical Engineering, College of Engineering and Physical Sciences, University of Birmingham UK

## Abstract

A comprehensive scientometric approach was adopted to study the research on ion exchange membranes. The statistical analysis was conducted based on 21 123 publications which were related to the topic of ion exchange membranes. Specifically, from 2001 to 2016, over 18 000 articles were published on ion exchange membranes, indicating researchers' great interest in this topic. Especially, compared to 2001, the number of articles published in 2016 increased approximately six-fold. This trend continued in 2017 since over 2000 articles were published in the year of 2017. Also, these articles were spread across over 1000 different journals, near 100 countries/regions and over 5000 research institutes, revealing the importance of ion exchange membrane as well as the broad research interest in this field. Besides, the properties and applications of ion exchange membranes were also discussed statistically. Furthermore, keywords analysis indicated that fuel cell and proton exchange membrane had the highest cooccurrence frequency. Finally, research areas analysis revealed that ion exchange membranes had a close relation with chemistry, energy and materials.

## Abstract 2

This paper provides a comprehensive statistical study on the topic of ion exchange membranes and may provide an avenue for future research work in this field.

## Graphical abstract

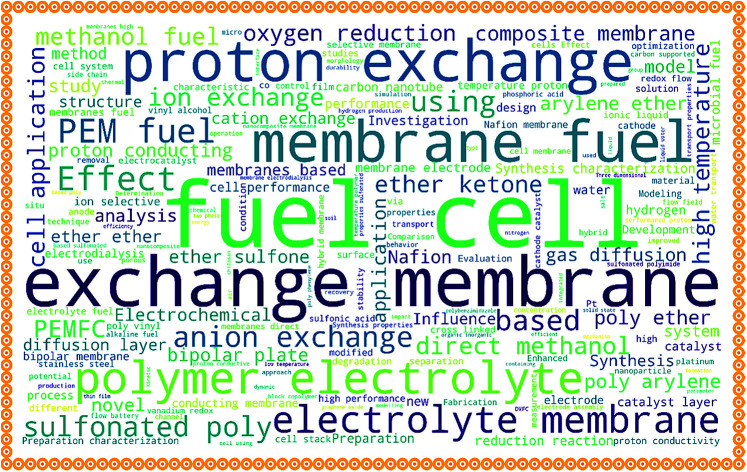

## 1. Introduction

Water and energy are the two biggest challenges in world today. A lot of effort has been made to address these two issues. Among the various methods and techniques developed or being developed, ion exchange membranes have demonstrated their capacity in dealing with these issues. Actually, ion exchange membranes are widely used in many applications, such as conventional fuel cells,^1^ microbial fuel cells,^2^ conventional electrodialysis,^3^ bipolar membrane electrodialysis,^4^ reverse electrodialysis,^5^ electrolysis for hydrogen production,^6^ redox flow batteries,^7^ diffusion dialysis,^8^ membrane capacitive deionization,^9^ and electrodeionization.^10^ Generally, ion exchange membranes are classified into two major types, namely cation exchange membranes and anion exchange membranes.^11–13^ Bipolar membrane is another kind of ion exchange membrane that combines cationic functional groups and anionic functional groups which are possessed by anion exchange membranes and cation exchange membranes, respectively.^14,15^ Proton exchange membrane, also known as polymer electrolyte membrane, is a common type of cation exchange membrane which is widely used in fuel-cell applications.^16,17^ Another special category of ion exchange membranes is named monovalent-ion-selective membranes, which, as indicated by its name, has the ability to separate monovalent ions from solutions and retain multivalent ions in solutions.^18^ For example, it can be used to remove arsenic and nitrate ions from groundwater,^19^ to concentrate reverse osmosis brines by electrodialysis,^20^ to generate energy through reverse electrodialysis,^21^ and so on. Besides, many different kinds of polymers have been investigated by researchers as potential ion exchange membranes *via* various synthesis methods.^22–24^ In a nutshell, the research in the field of ion exchange membranes is prosperous.

Meanwhile, in the age of big data, a lot of useful information can be obtained through data analysis. Therefore, instead of making decisions based solely on subjective experience, we can make decisions more scientifically with the help of data analysis. In other words, we can now conduct quantitative analysis as an insightful supplement to conventional qualitative analysis or review. As discussed above, a lot of work has been done on the topic of ion exchange membranes. Given researchers' great interest, it is necessary and important to give a timely update on this field so readers will not get lost in front of thousands of new papers published every year in this field. Although quite a few review papers have been published on the topic of ion exchange membranes, they are mainly based on the authors' subjective experience. There are very limited, if not zero, number of publications on the topic of ion exchange membranes using comprehensive statistical approach, or quantitative data analysis. Now the question is, how to carry out quantitative analysis on ion exchange membranes? Scientometrics is a quantitative study of the progress of science and scientific research,^25^ and is being widely used by researchers in various fields, such as sustainability,^26^ building information modelling,^27^ antibiotics,^28^ and so on. This paper aims to provide a comprehensive statistical study on the topic of ion exchange membranes through scientometric approach.

## 2. Materials and methods

The data used in this study was obtained from the database of Web of Science Core Collection with time span of 2001–2016, with citation index of Science Citation Index Expanded (SCI-EXPANDED) and with the following search terms for topic:

(“*ion* exchange* membrane*” or “*bipolar membrane*” or “proton* exchange* membrane*” or “*polymer* electrolyte* membrane*” or “*ion* *selective membrane*” or “*ion* conduct* membrane*” or “proton* conduct* membrane*” or “*ion* exchange* film*”)

Examples of search terms were listed in Table 1. For example, “*ion* exchange* membrane*” included terms like “ion exchange membrane”, “ion exchange membranes”, “cation exchange membrane”, “anionic exchange membrane”, and so on. A total of 21 123 publications met this search criteria. Full record of these publications was downloaded as .txt files and analyzed by software programs including Microsoft Excel, SPSS, ATLAS.ti, BibExcel, and Gephi. As an alternative, python programming skills can be helpful to process the results. The parts of full record data studied included title, address, publication name, language, document type, keywords, abstract, citations, yeas published, and research areas. Raw data was provided in Data repository and detailed data processing procedures were provided in ESI.†

**Table 1:** Examples of search terms for topic

Search term	Separate term	Examples of separate term
“*Ion* exchange* membrane*”	*Ion*	Ion, cation, anion, ionic, cationic, anionic
	Exchange*	Exchange, exchanger, exchangeable, exchanged
	Membrane*	Membrane, membranes
“*Bipolar membrane*”	*Bipolar	Bipolar, ambipolar
	Membrane*	Membrane, membranes
“Proton* exchange* membrane*”	Proton*	Proton, protonic
	Exchange*	Exchange, exchanger, exchangeable, exchanged
	Membrane*	Membrane, membranes
“*Polymer* electrolyte* membrane*”	*Polymer*	Polymer, copolymer, polymeric
	Electrolyte*	Electrolyte, electrolytes
	Membrane*	Membrane, membranes
“*Ion* *selective membrane*”	*Ion*	Ion, cation, anion, ionic, cationic, anionic
	*Selective	Selective, permselective
	Membrane*	Membrane, membranes
“*Ion* conduct* membrane*”	*Ion*	Ion, cation, anion, ionic, cationic, anionic
	Conduct*	Conducting, conductive
“Proton* conduct* membrane*”	Proton*	Proton, protonic
	Conduct*	Conducting, conductive
	Membrane*	Membrane, membranes
“*Ion* exchange* film*”	*Ion*	Ion, cation, anion, ionic, cationic, anionic
	Exchange*	Exchange, exchanger, exchangeable, exchanged
	Film*	Film, films

## 3. Results and discussion

### 3.1 Document types

**Fig. 1:**
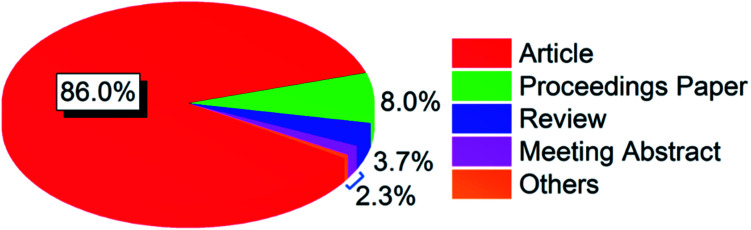
Percentage distribution of document types.

The publications were divided into 10 document types, where article was the dominating type accounting for 86.0% of the total. As shown in Fig. 1, the next two types were proceedings paper and review, which had a percentage of 8.0% and 3.7%, respectively. The fourth document type was meeting abstract, which accounted for 1.7% of the total. The remaining six document types with a total percentage of 0.6% were correction, book chapter, editorial material, letter, news item, and retracted publication, respectively. The following analysis was based on articles only since it was the main document type with a total number of 18 166.

### 3.2 Publishing languages

**Fig. 2:**
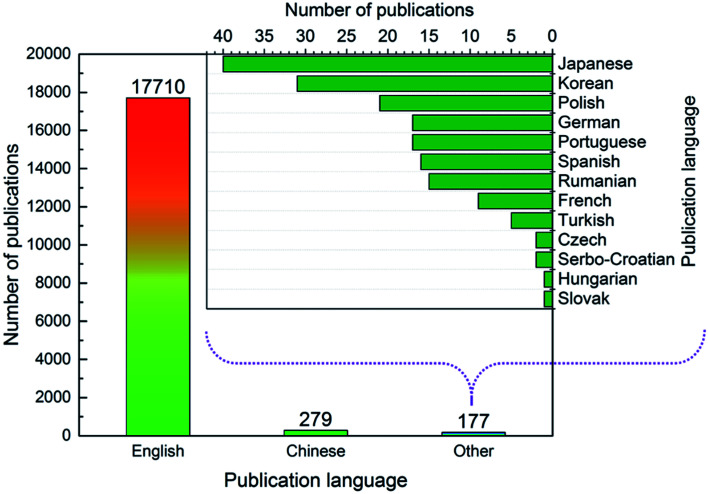
Number of publications in different languages.

As shown in Fig. 2, most articles were published in English (97.5%), followed by Chinese (1.5%). Articles published in other languages (*e.g.*, Japanese, Korean) made up only 1.0% of the total articles. Given that the most common publishing journals for ion exchange membrane research were English-language journals (see 3.4), it was expected that English was the most common publication language.

### 3.3 Publishing trend

**Fig. 3:**
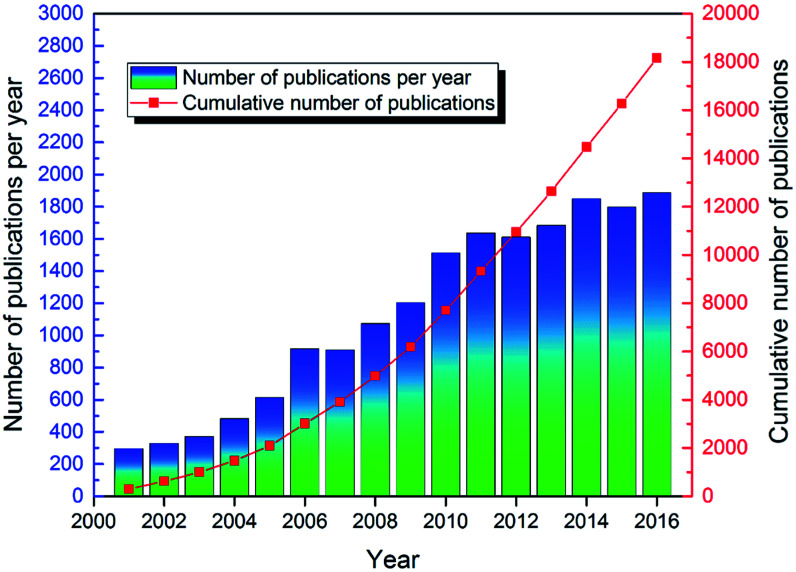
Number of publications per year and cumulative number of publications on ion exchange membranes since 2001.

As shown in Fig. 3, from 2001 to 2016, over 18 000 articles were published on the topic of ion exchange membranes, indicating researchers' great interest in this topic. Specifically, compared to 2001, the number of articles published in 2016 increased approximately six-fold. From 2001 to 2011 there was a rapid increase in the annual number of articles published on this topic. Then, the speed slowed down. From 2011 to 2013, and from 2014 to 2016, the increase in number of annual publications seemed to reach a plateau around 1600 and 1800, respectively. This might suggest that the early 2000s was a period of rapidly evolving research on ion exchange membranes that stabilized somewhat recently. Despite this, the topic of ion exchange membranes is still an active research topic given that since 2011, over 1600 articles were published annually. This trend continued in 2017 since over 2000 articles were published in the year of 2017. Actually, in recent years, there were some quite exciting and new studies in the field of ion exchange membranes, which were discussed in detail in the following sections.

### 3.4 Publishing journals

**Fig. 4:**
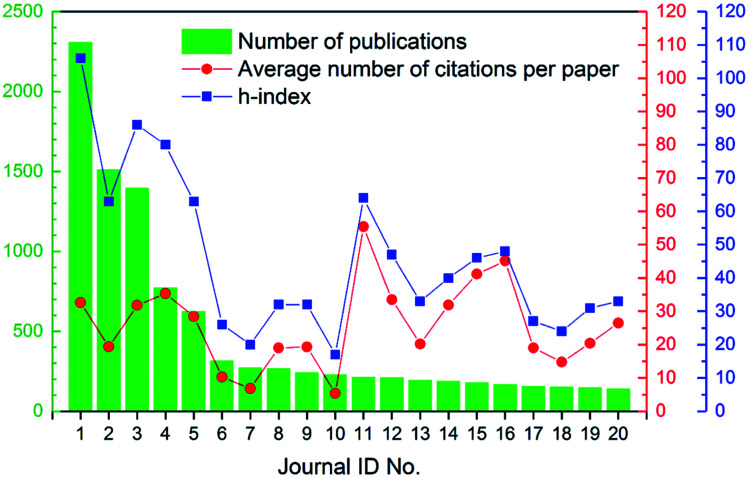
Number of publications, average number of citations per paper, and h-index of the top 20 most publishing journals.

The 18 166 articles in this study were spread across 1092 different journals, which indicated the popularity and diversity of this research field. On the other hand, 80.0% of the journals had eight or fewer articles published on the topic of ion exchange membranes (see Fig. S1 in ESI†). The top twenty most publishing journals were summarized in Table 2. Interestingly, these twenty journals accounted for 53.3% of all articles published in the 16 year time span. Besides, as revealed by Table 2, Elsevier, American Chemical Society (ACS), and Royal Society of Chemistry (RSC) were the top three most common publishers. As shown by Fig. 4, in general, h-index was higher than average number of citations per paper. But the really interesting thing was that Fig. 4 revealed a positive correlation between h-index and citations, which was comprehensible as h-index was a composite of the number of papers and citations (namely impact). To sum up, as revealed by the journals analysis, the field of ion exchange membranes was quite popular and diversified, but it did have its own focused-areas. In fact, as discussed below, ion exchange membranes had many important applications, with water and energy being the two most concerned ones.

**Table 2:** Top 20 most publishing journals^a^

ID No.	Journal name	Publisher
1	Journal of Power Sources	Elsevier
2	International Journal of Hydrogen Energy	Elsevier
3	Journal of Membrane Science	Elsevier
4	Journal of the Electrochemical Society	ECS
5	Electrochimica Acta	Elsevier
6	Journal of Applied Polymer Science	John Wiley & Sons
7	RSC Advances	RSC
8	Fuel Cells	John Wiley & Sons
9	Desalination	Elsevier
10	Journal of Fuel Cell Science and Technology	ASME
11	Macromolecules	ACS
12	Polymer	Elsevier
13	Journal of Materials Chemistry A	RSC
14	Journal of Physical Chemistry C	ACS
15	Journal of Physical Chemistry B	ACS
16	Electrochemistry Communications	Elsevier
17	Solid State Ionics	Elsevier
18	Separation and Purification Technology	Elsevier
19	ACS Applied Materials & Interfaces	ACS
20	Physical Chemistry Chemical Physics	RSC

a ECS refers to Electrochemical Society; RSC refers to Royal Society of Chemistry; ASME refers to American Society of Mechanical Engineers; ACS refers to American Chemical Society.

### 3.5 Publishing countries/regions

**Fig. 5:**
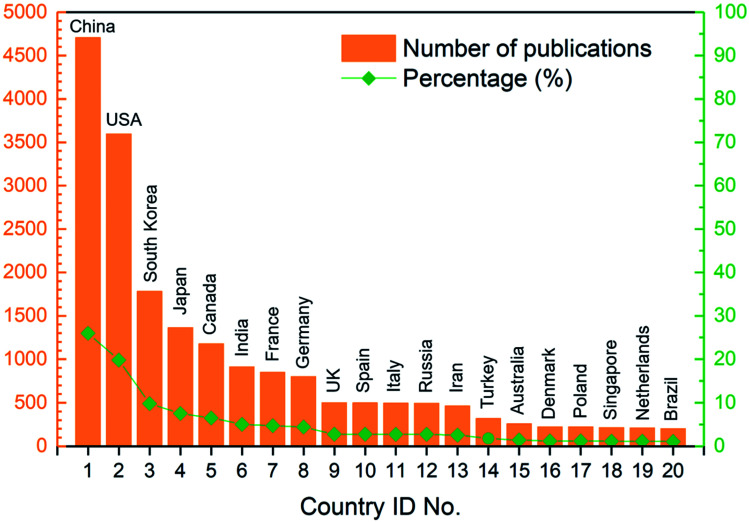
Number of publications and corresponding percentage of the top 20 most publishing countries.

**Fig. 6:**
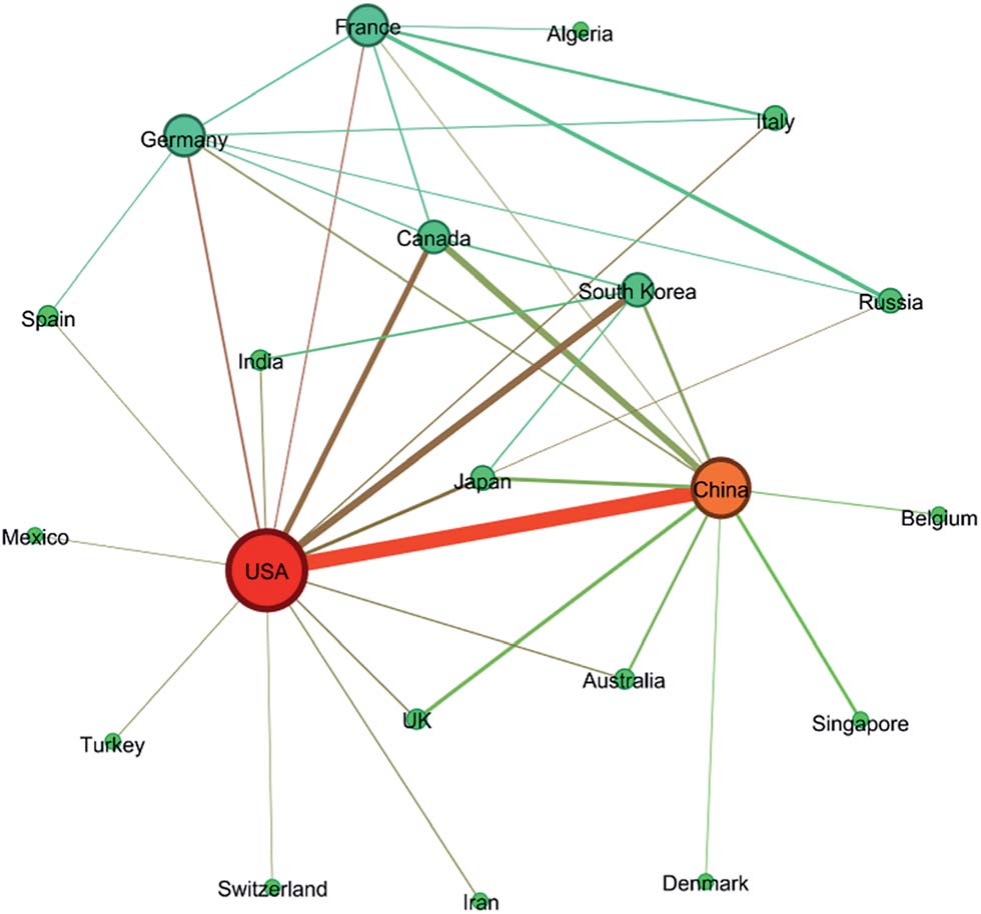
Network graph showing collaborations between countries. Countries with collaborations of more than 20 times were connected with lines.

In general, 98 countries/regions in total had contributions to these articles, which revealed that the field of ion exchange membranes was of global interest. Among these countries/regions, 40 countries/regions published more than 40 articles and 30 countries/regions published more than 100 articles during the 16 year period (see Table S2 in ESI†). As revealed by Fig. 5, China and USA were the top two most publishing countries. The following three countries whose article number exceeded 1000 were South Korea, Japan and Canada. On the other hand, 17.28% of the articles were published as a result of collaborations between two or more countries/regions. As shown in Fig. 6, around 40 pairs of countries published more than 20 articles together, where the USA/China pair had the most fruitful collaborations (also see Table S4 in ESI†).

### 3.6 Publishing institutions

**Fig. 7:**
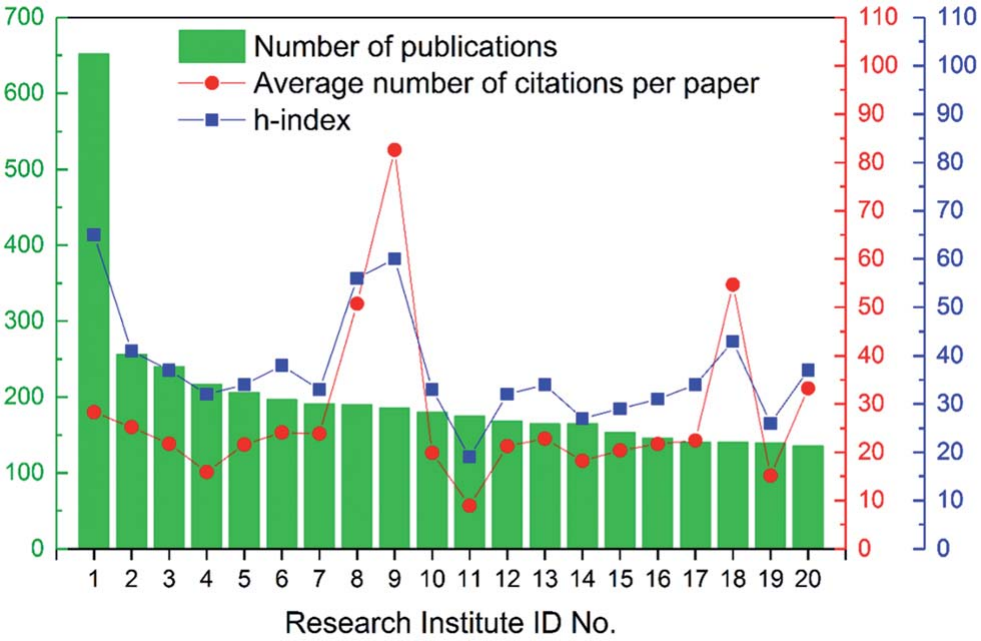
Number of publications, average number of citations per paper, and h-index of the top 20 most publishing research institutes.

In general, over 5000 research institutes had contributions to these articles, which again uncovered the broad research interest in the field of ion exchange membranes. Specifically, among these institutes, 34 institutes published 100 articles or more during the 16 year period (see Table S5 in ESI†). As shown in Fig. 7 and Table 3, Chinese Academy of Sciences was the no. 1 most publishing research institute (over 650 articles), followed by University of Science and Technology of China. From another perspective, it should be pointed out that institutes like Chinese Academy of Sciences, Indian Institutes of Technology, and so on are centrally funded clusters of research institutes, which enabled their high production. In other words, the institutes listed in Table 3 can be roughly divided into two groups, namely independent universities such as Jilin University and “institute clusters” such as Chinese Academy of Sciences. Actually, there are quite a few research centres/groups in Chinese Academy of Sciences that focus on the field of ion exchange membranes as well as membrane applications, including but not limited to fuel cells, electrodialysis and electrolysis.

**Table 3:** Top 20 most publishing research institutes

ID No.	Research Institute
1	Chinese Academy of Sciences
2	University of Science and Technology of China
3	Jilin University
4	Shanghai Jiao Tong University
5	Seoul National University
6	Tsinghua University
7	Korea Institute of Science and Technology
8	National Research Council Canada
9	Pennsylvania State University
10	Wuhan University of Technology
11	Russian Academy of Sciences
12	Indian Institutes of Technology
13	Hanyang University
14	Tianjin University
15	Yuan Ze University
16	Tokyo Institute of Technology
17	Dalian University of Technology
18	University of South Carolina
19	Kuban State University
20	University of Connecticut

**Fig. 8:**
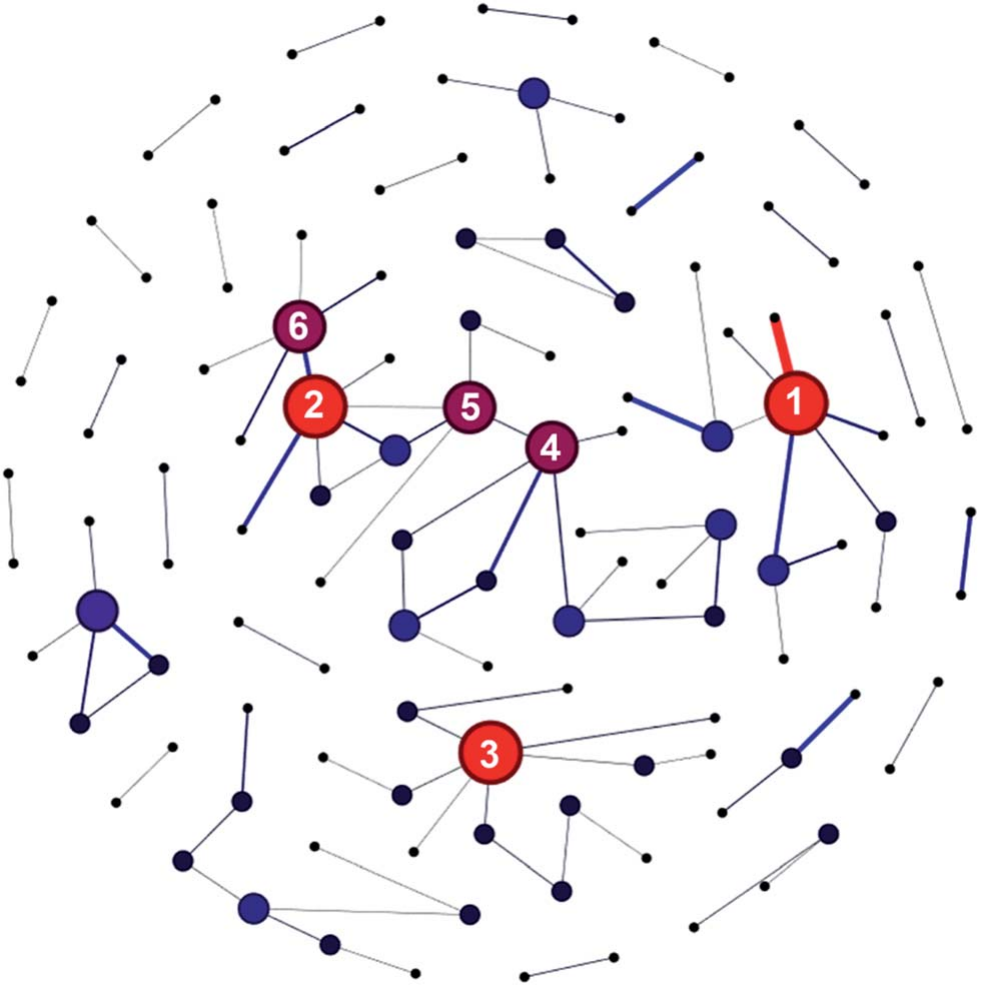
Research institute collaboration network graph. Institutes whose collaborations exceeded 10 times were connected with lines. Note: (1) Chinese Academy of Sciences; (2) Korea Institute of Science and Technology; (3) National Taiwan University; (4) National Research Council Canada; (5) Hanyang University; (6) Seoul National University.

Besides, 48.13% of the articles were published as a result of collaborations between two or more research institutes. Furthermore, there were around 1000 pairs of research institutes that published two or more articles together. Chinese Academy of Sciences and University of Chinese Academy of Sciences had the most fruitful collaborations with each other (see Table S7 in ESI†). As revealed by Fig. 8, Chinese Academy of Sciences, Korea Institute of Science and Technology, and National Taiwan University ranked top three in terms of number of collaborating institutes. To be specific, take Chinese Academy of Sciences for example, it had collaborations with 6 different research institutes and published more than 10 articles together with each of these six institutes.

### 3.7 Most-cited papers

The top twenty most-cited articles on the topic of ion exchange membranes since 2010 were summarized in Table 4, including total citations, year published as well as research direction. It should be pointed out that the research directions were not necessarily mutually exclusive. For example, direct formic acid fuel cell (DFAFC) was a subcategory of proton exchange membrane fuel cell or polymer electrolyte membrane fuel cell (PEMFC). Among the articles listed in Table 4, it seemed that although ion exchange membranes had many applications, fuel cells were the most studied ones, which corresponded well with the following title and abstract analysis.

**Table 4:** Top 20 most-cited articles^a^

No.	Articles	TC	PY	Research category
1	V. R. Stamenkovic^29^	2096	2007	PEMFC
2	B. Lim *et al.*^30^	1811	2009	PEMFC
3	M. Lefèvre *et al.*^31^	1512	2009	PEMFC
4	J. Greeley *et al.*^32^	1118	2009	PEMFC
5	G. Nagel *et al.*^33^	1083	2003	CSM
6	H. Liu *et al.*^34^	1017	2004	MFC
7	F. Wang *et al.*^35^	868	2002	PEMFC
8	E. J. Popczun *et al.*^36^	859	2013	HER
9	D. B. Levin *et al.*^37^	816	2004	PEMFC
10	P. J. Ferreira *et al.*^38^	781	2005	PEMFC
11	K. Schmidt-Rohr *et al.*^39^	706	2008	PEMFC
12	C. G. Van de Walle *et al.*^40^	666	2003	PEMFC^b^
13	J. H. Nam *et al.*^41^	627	2003	PEMFC
14	C. Wang *et al.*^42^	625	2004	PEMFC
15	P. Xing *et al.*^43^	622	2004	PEMFC
16	Z. H. Wang *et al.*^44^	609	2001	PEMFC
17	B. Logan *et al.*^45^	603	2007	MFC
18	S. Cheng *et al.*^46^	594	2006	MFC
19	S. M. Haile *et al.*^47^	591	2001	SAFC
20	C. Rice *et al.*^48^	537	2002	DFAFC

a TC refers to total citations; PY refers to publishing year; PEMFC refers to proton exchange membrane fuel cell or polymer electrolyte membrane fuel cell; CSM refers to cation selective membrane; MFC refers to microbial fuel cell; SAFC refers to solid acid fuel cell; HER refers to hydrogen evolution reaction; DFAFC refers to direct formic acid fuel cell.

b The more direct research topic of this paper was hydrogen, understanding of which is important for PEMFC development.

### 3.8 Title analysis

**Fig. 9:**
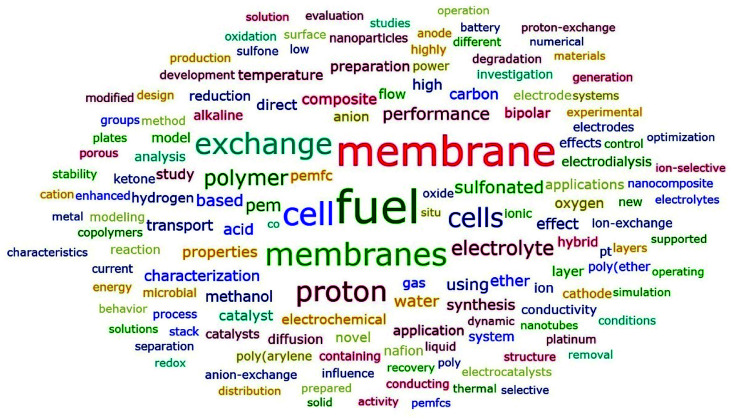
Word cloud generated from titles with frequency no less than 200.

As shown in Fig. 9, fuel was the most common word in titles, which had a frequency of 7515, meaning that over 40% of the articles contained the word “fuel” in their titles. Following fuel were membrane, cell and membranes, which ranked 2^nd^, 3^rd^, and 4^th^, respectively. There was no surprise that the two words “membrane” and “membranes” had top rankings in terms of frequency. A further analysis revealed that 7350 articles out of the 18 166 articles contained fuel cell or fuel cells, which indicated that fuel cell(s) was the most frequently studied subfield in the field of ion exchange membranes. The following two were exchange and proton. Similarly, a further analysis showed that 2901 articles contained proton exchange membrane or proton exchange membranes, which indicated that proton exchange membrane(s) was the most frequently studied membrane type of ion exchange membranes. Polymer and electrolyte were another two common words appearing in the titles. A further analysis revealed that polymer electrolyte membrane(s) was contained in the titles of 1404 articles. In fact, polymer electrolyte membrane fuel cells (PEMFCs) was often used as a whole. Furthermore, 141 titles contained cation exchange membrane(s) while 517 titles contained anion exchange membrane(s). However, this did not necessarily mean that cation exchange membranes were less popular compared to anion exchange membranes. Another noteworthy high-frequency word was “sulfonated”, which was contained in the titles of 1624 articles out of the 18 166 articles. Given the high frequency of proton exchange membrane(s) which was a kind of cation exchange membranes, it was understandable that sulfonate also had a high frequency as well, since sulfonate group was the most common functional group for cation exchange membranes. On the other hand, it indicated that a lot of studies on sulfonated polymers had been done, which was further verified by the fact that 1252 articles contained “sulfonated poly”, among which sulfonated poly(ether ether ketone) (SPEEK) was the most studied one (Nafion was excluded from this comparison and was analyzed specifically below).

### 3.9 Abstract analysis

**Fig. 10:**
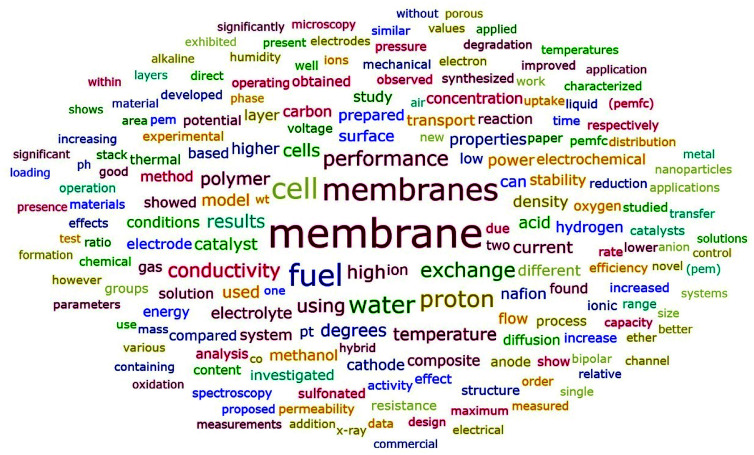
Word cloud generated from abstracts with frequency no less than 1000.

Fig. 10 showed similar frequency distributions as Fig. 9. The word “water” appeared more frequently in the abstracts than in the titles. To be specific, “water” was the fifth most common word in abstracts, which appeared in 6689 abstracts, revealing the important relationship between water and ion exchange membranes. As shown in Table 5, fuel cell was the most common application of ion exchange membranes, followed by electrodialysis. As revealed by Fig. 10 and Table 6, conductivity was the most studied membrane property, indicating its important position in ion exchange membranes. Besides, stability was another most studied membrane property. In order to make membranes commercially feasible and more competitive, it is crucial that excellent membrane stability is guaranteed. Furthermore, the word “Nafion” appeared 3143 times in these abstracts, meaning that 17.40% of the abstracts contained “Nafion”, which indicated its important role in the field of ion exchange membranes. Nafion is a sulfonated tetrafluoroethylene based perfluorinated polymer^49^ which is widely used in many applications such as fuel cells.^50^ Other common studied polymers included poly(ether ether ketone) (PEEK), polybenzimidazole (PBI), and so on (see Table S8 in ESI† for more details).

**Table 5:** Common applications of ion exchange membranes^a^

Application	Number of abstracts
Fuel cell	10 261
Electrodialysis	843
Electrolysis	447
Desalination	256
Redox flow battery	225
Bipolar membrane electrodialysis	182
Diffusion dialysis/Donnan dialysis	157
Water treatment	104
Reverse lectrodialysis	82
Capacitive deionization	52
Electrodeionization	43
Water purification	31
Electro-electrodialysis	25

a The applications listed in this table were not necessarily mutually exclusive.

**Table 6:** Common properties of ion exchange membranes^a^

Property	Number of abstracts
Conductivity	4952
Stability	3301
Water uptake	2175
Resistance	2158
Ion exchange capacity	1600
Permeability	1465
Morphology	1150
Thermal stability	1145
Swelling	1033
Hydrophilic	979
Hydrophobic	874
Mechanical properties	833
Chemical properties	519
Chemical stability	466
Oxidative stability	337
Dimensional stability	306

a The properties listed in this table were not necessarily mutually exclusive.

From another perspective, the above analysis is mainly focused on the statistical aspect of ion exchange membranes. In other words, it is powerful in revealing the “major trend” or “broad interest” of this field but is less powerful to reveal the latest research breakthroughs or issues. Therefore, it is important to give a “human-knowledge-based” analysis on this topic, as a supplement. For example, bipolar membrane is gaining more research interest in recent years due to its high energy-efficiency as well as environmental benefits. One special research interest is bipolar membrane water splitting.^51^ In addition to conventional preparation methods (*e.g.*, casting technique), researchers are also exploring other novel technologies, such as electrospinning to control the layer thickness precisely.^52^ Another very promising topic is water electrolysis membranes. This technology is gaining growing interest due to its capacity to produce hydrogen from cheap and readily available resources, *i.e.*, water. However, it does not mean the hydrogen production process is cheap. So one research interest is to reduce high capital cost as well as uncertainty related to this process.^53^ Besides, another interesting aspect of water electrolysis membranes is product gas crossover.^54^ Finally, researchers are also developing new membranes, including mixed matrix membranes,^55^ short side chain perfluorosulfonic acid membranes,^56^ and so on. However, more work needs to be done to make these membranes more robust. For example, the mechanical stabilities of mixed matrix membranes could be a problem due to the addition of particles.

### 3.10 Keywords analysis

**Fig. 11:**
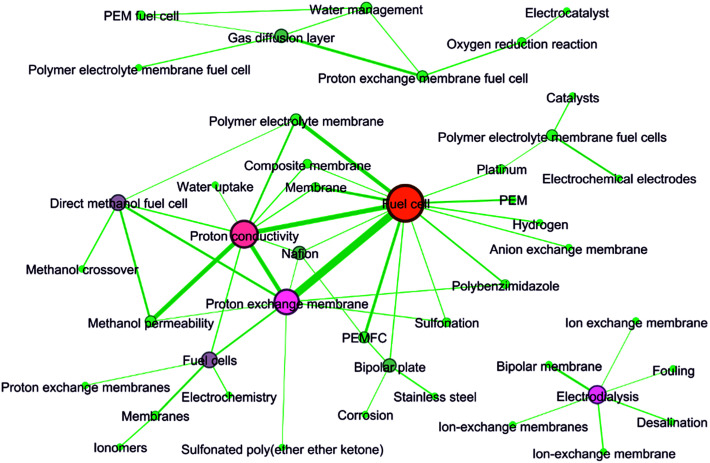
Keywords network graph. Keywords whose cooccurrence exceeded 30 times were connected with lines.

As revealed by Table 7, Fuel cell was obviously the keyword with highest frequency. This result agreed well with the above discussions. In brief, the development of ion exchange membranes was mainly driven by two worldwide issues/concerns, *i.e.* energy and water. Fuel cell was on the energy side. Besides, Fuel cell and Proton exchange membrane had the highest cooccurrence frequency, followed by Fuel cell and Proton conductivity. Interestingly, as shown in Fig. 11, there were three independent keywords networks. The Fuel cell-centered network and the Electrodialysis-centered network revealed the two most studied applications of ion exchange membranes. This result was consistent with the analysis from the previous sections.

**Table 7:** Top 20 most used keywords for ion exchange membranes

Keyword	Number of articles	Percentage (%)	Rank
Fuel cell	1386	9.86	1
PEMFC	824	5.86	2
Proton exchange membrane	789	5.61	3
Proton exchange membrane fuel cell	743	5.29	4
Fuel cells	709	5.04	5
Proton conductivity	699	4.97	6
PEM fuel cell	575	4.09	7
Electrodialysis	504	3.59	8
Membranes	369	2.63	9
Proton exchange membrane fuel cells	346	2.46	10
Polymer electrolyte membrane	326	2.32	11
Nafion	325	2.31	12
Membrane	323	2.30	13
Direct methanol fuel cell	315	2.24	14
Polymer electrolyte membrane fuel cell	310	2.21	15
Anion exchange membrane	307	2.18	16
Oxygen reduction reaction	297	2.11	17
Gas diffusion layer	270	1.92	18
PEM fuel cells	232	1.65	19
Durability	222	1.58	20

### 3.11 Research areas analysis

**Fig. 12:**
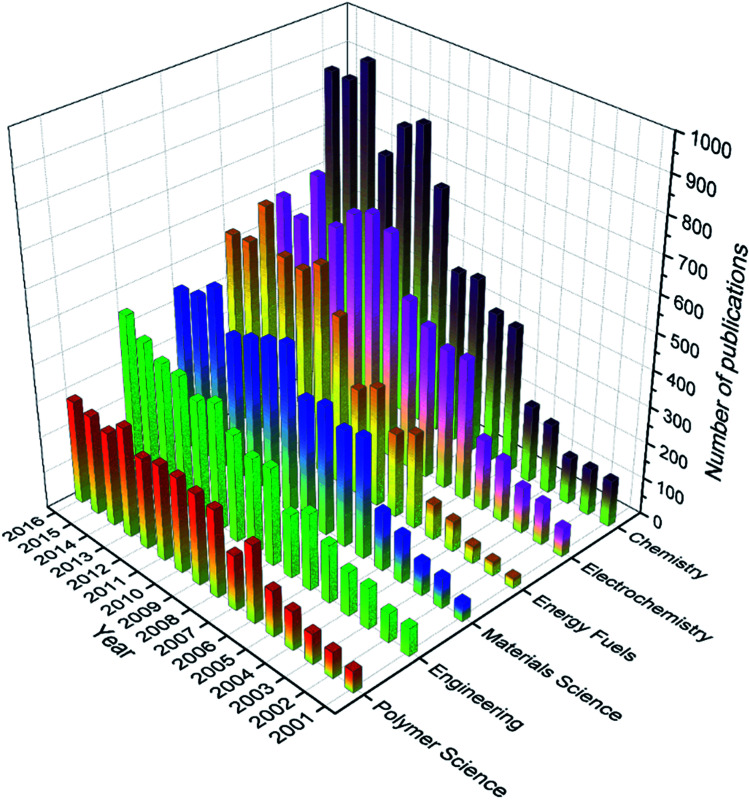
Number of publications in the top six research areas every year from 2001 to 2016.

The research areas discussed in this section referred to the categories in the SC attribute in the database where SC was short for research areas as defined by Web of Science. The 18 166 articles were categorized into 68 research areas, and these research areas appeared 37 873 times in total, meaning that one article were categorized into two research areas on average. Specifically, 18 of the 68 research areas had 100 articles or more, and only 8 out of the 68 research areas had 1000 articles or more (see Fig. S2 in the ESI†). Interestingly, there seemed to be a big gap between the 8^th^ research area and the 9^th^ research area, where the former had more than 1000 articles while the latter had less than 500 articles. Moreover, from 1^st^ to 8^th^, the number of publications decreased very quickly compared to that from 8^th^ to the last one. The most common research area was Chemistry, and 8227 articles were categorized into this area. In other words, 45.3% of the total articles were categorized into Chemistry. The next top five research areas were Electrochemistry (36.6%, 2^nd^), followed by Energy & Fuels (29.2%, 3^rd^), Materials Science (27.7%, 4^th^), Engineering (22.3%, 5^th^), and Polymer Science (17.6%, 6^th^). As revealed by Fig. 12, almost all of these research areas (except Engineering) seemed to reach a plateau, some of which even showed a tendency to decrease. These trends were generally in line with the overall publication trend discussed in 3.3.

## 4. Conclusion

A comprehensive statistical study on the research of ion exchange membranes was conducted *via* a scientometric approach. Based on statistical analysis of the 21 123 publications which were related to the topic of ion exchange membranes, it was found that article was the dominating type for these publications and accounted for 86.0% of the total. English was the dominating publishing language for these articles. Specifically, from 2001 to 2016, over 18 000 articles were published on ion exchange membranes, indicating researchers' great interest in this topic. Especially, compared to 2001, the number of articles published in 2016 increased approximately six-fold. This trend continued in 2017 since over 2000 articles were published in the year of 2017. Also, these articles were spread across over 1000 different journals, near 100 countries/regions and over 5000 research institutes, revealing the importance of ion exchange membrane as well as the broad research interest in this field. Besides, the properties and applications of ion exchange membranes were also discussed statistically. Furthermore, keywords analysis indicated that fuel cell and proton exchange membrane had the highest cooccurrence frequency. Finally, research areas analysis revealed that ion exchange membranes had a close relation with chemistry, energy and materials. To conclude, this scientometric study provides a statistical analysis on ion exchange membranes and may provide an avenue for future research work in this field.

## Data repository

All raw data used in this study is available from the open access repository: https://doi.org/10.5281/zenodo.1227129.

## Conflicts of interest

The authors declare no conflict of interest.

## Supplementary Material

RA-008-C8RA04686G-s001
