# Supplementary material for: A scientometric study of the research on ion exchange membranes
Source: RSC Adv. 2018 Jul 2;8(42):24036–48. doi: 10.1039/c8ra04686g (PMC9081738; doi:10.1039/c8ra04686g)
Supplement: RA-008-C8RA04686G-s001 [file RA-008-C8RA04686G-s001.pdf]

Electronic Supplementary Material (ESI) for RSC Advances.
This journal is © The Royal Society of Chemistry 2018

Supplementary Information

A scientometric study of the research on ion exchange membranes

Shanxue Jiang[a], Kimberly F L Hagesteijn[a], Jin Ni[b], Bradley P Ladewig[a,*]

[a]*Barrer Centre, Department of Chemical Engineering, Imperial College London, United Kingdom*
[b]*School of Chemical Engineering, College of Engineering and Physical Sciences, University of Birmingham, United Kingdom*

---

1. Data Processing Procedures

The data was downloaded from Web of Science in December 2017. After importing all the data from .txt files into Excel, the data was further processed. First and foremost, there were many columns containing different types of information. Therefore, the columns used in this study were picked out and further analysed, including TI, SO, LA, DT, DE, AB, C1, TC, PY, SC. The meaning of these abbreviations was summarized in Table S1.

Table S1: Abbreviations and corresponding meaning

**Abbreviation**	**Meaning**
TI	Title
SO	Publishing Journal
LA	Language
DT	Document Type
DE	Author Keywords
AB	Abstract
C1	Addresses
TC	Total Citations
PY	Publishing Year
SC	Research Areas

Then, the following three modifications were made using the Find & Replace function in Excel to eliminate punctuation differences that might affect data analysis for the overall data set. The first modification was to remove the space after comma. The second modification was to remove the space after semicolon. The third modification was to remove the double quotes. Further data processing was discussed in the respective sections below. Overall, one citation was removed due to format issues with the imported citation.

1.1. Document Type (DT) processing

Using the DT data column, a Pivot Table was created in Excel to determine the count and percentage of the different document types. If a citation had multiple document types selected (e.g. Article; Proceedings Paper), it was classified into the document type that was the best fit.

*Corresponding author
Email address: `b.ladewig@imperial.ac.uk` (Bradley P Ladewig)

1.2. Language (LA) processing

LA processing was similar to DT processing. Using the LA data column, a Pivot Table was created in Excel to determine the count and percentage of each journal article publication language. One citation was listed as both Chinese and English, but was amended to Chinese after reading the paper.

1.3. Publishing Year (PY) processing

Similarly, using the PY data column, a Pivot Table was created in Excel to determine the count and percentage of the number of journal articles published per year.

1.4. Publishing Journal (SO) processing

Similarly, using the SO data column, a Pivot Table was created in Excel to determine the count and percentage of the number of journal articles published in different journals.

1.5. Total Citations (TC) processing

As is known, citations for a paper tend to increase as time goes on. Since the data was downloaded in December 06, 2017, the total citations were updated to this date as well.

For a specific journal, its average number of citations per paper was calculated by dividing the total citations by number of articles published in this journal. The h-index of a journal used in this study referred to the highest number of articles published in this journal which had h or more citations each while the other articles had less than h citations each. Besides, the h-index was calculated based on articles published since 2001. Also, the h-index was calculated based on the data was downloaded in December 06, 2017. Therefore, the more accurate expression of h-index would be h-index since 2001 by December 06, 2017. The above method was also adopted in calculating the h-index and average number of citations per paper of the research institutes.

1.6. Addresses (C1) processing

In general, C1 processing was used to analyse publishing research institutes and publishing countries/regions.

1.6.1. Basic processing

Column C1 contained institution addresses which were used to acquire research institutes and countries/regions. Out of the 18166 articles, 18151 articles contained address information in Column C1 and therefore constituted the samples for acquiring research institutes and countries/regions. Column C1 was then refined to remove square brackets, the content in the square brackets as well as the blank space following the square brackets. This was realized through the Replace tool in Excel. For example, before processing, the content in C1 of an article sample was read as follows:

[Li,Q.;He,R.;Jensen,J. O.;Bjerrum,N. J.] Tech Univ Denmark,Dept Chem,DK-2800 Lyngby,Denmark

After processing, the content in C1 of this article sample was read as follows:

Tech Univ Denmark,Dept Chem,DK-2800 Lyngby,Denmark

Also, an article could have one or more institution addresses. These addresses were originally contained in one cell for one article. Therefore, the C1 column was separated into several columns so that one address was contained in one cell. This was realized through the Text To Columns tool in Excel, using semicolon as the delimiter. For example, before processing, the cell content of an article sample was read as follows:

Northeastern Univ,Dept Chem & Chem Biol,Boston,MA 02115 USA;Univ Texas Rio Grande Valley,Dept Phys,Edinburg,TX 78539 USA

After processing, the cell content of this article sample was separated into two cells, which were read as follows:

In one cell:

Northeastern Univ,Dept Chem & Chem Biol,Boston,MA 02115 USA

In the other cell:

Univ Texas Rio Grande Valley,Dept Phys,Edinburg,TX 78539 USA

Further, the addresses were refined twice independently, one was to keep the institution name only while the other was to keep country/region only. This was realized through the Replace tool in Excel. For example, before processing, the cell content of an article sample was read as follows:

Murdoch Univ,Sch Environm Sci,Murdoch,WA 6150,Australia

To keep the institution name only, the content after the first comma (including the first comma) was replaced with blank, and therefore the cell content was changed to:

Murdoch Univ

Similarly, to keep the country name only, the content before the last comma (including the last comma) was replaced with blank, and therefore the cell content was changed to:

Australia

As is known, an article could have two or more institution addresses with the same institution name. Therefore, the next step was to remove the repetitive institution names in each article. This was realized through the MATCH function together with the Filter tool in Excel. For example, before processing, an article sample had four institution names, shown as follows:

Univ Illinois Univ Illinois Univ Illinois Carnegie Mellon Univ

After processing, this article sample had two institution names, shown as follows:

Univ Illinois Carnegie Mellon Univ

Then, the Pivot Table tool in Excel was used to determine the count of each institution and each country. The RANK function in Excel was used to calculate the rank of each research institute and country in terms of articles number.

It should be pointed out that occasionally two research institutes, which were actually the same institute, were stored in the database under different names. For example, Tsinghua Univ and Tsing Hua Univ. It was really time-consuming and difficult to find all these "bugs". On the other hand, considering the large sample size in this study, most of these "bugs" would not have a noticeable effect on the results. Therefore, only the top ranking research institutes were carefully scrutinized to prevent or minimize possible effects. To be specific, Tsinghua Univ and Tsing Hua Univ were actually the same institution, and therefore were put together as Tsinghua Univ in this study. Besides, in this study, Chinese Acad Sci YICCAS and Chinese Acad Sci were regarded as two institutes.

1.6.2. Network graph processing

Figure 5, 7, and 10 were network graphs. The software program named Gephi was used to generate these network graphs. For detailed instructions on using it, please visit its official website at https://gephi.org/. However, before using Gephi, another software program named BibExcel was needed to general the pairs data information which was then used for Gephi.

1.7. Title (TI) processing

TI column was used to create word cloud based on titles. To begin with, the TI column was saved as .txt file. Then the double quotes in the .txt file was removed via the Replace tool. Then, this file was imported into the software ATLAS.ti. Then word cloud could be generated using the Word Cloud function. It should be pointed out that the common words like a, an, the were automatically screened out by this software program.

1.8. Abstract (AB) processing

Among the 18166 articles, 18060 contained abstracts. And the following analysis in this part was based on these abstracts.

1.8.1. Word cloud generation

Similarly, AB column was used to create word cloud based on abstracts. To begin with, as AB column contained copyright information (see the following copyright examples), it needed to be removed as it was not related to this study. This was realized through the Replace tool in Excel.

Copyright examples:

(C) 2004 Elsevier B.V. All rights reserved.
(C) 2004 Elsevier Ltd. All rights reserved.
(C) 2004 Elsevier Inc. All rights reserved.
(C) 2004 International Association for Hydrogen Energy. Published by Elsevier Ltd. All rights reserved.
(C) 2003 Silsoe Research Institute. All rights reserved Published by Elsevier Ltd.
(C) 2008 Published by Elsevier B.V.

Then the double quotes in the .txt file was removed via the Replace tool. Then, this file was imported into the software ATLAS.ti. Then word cloud could be generated using the Word Cloud function.

1.8.2. Applications of ion exchange membranes

The results in Table 6 were based on the abstracts analysis which was done using the Filter tool in Excel. The following *"?"* was used to represent any single character. The numbers had the following meanings.

- 10261 abstracts contained "fuel cell" or "fuel-cell".
- 843 abstracts contained "electrodialysis" or "electro?dialysis", where "electro?dialysis" included "electro dialysis" and "electro-dialysis". 580 abstracts out of the 843 abstracts did not contain "reverse?electrodialysis" and "bipolar membrane". It meant that, 580 abstracts mentioned electrodialysis but did not mention bipolar membrane and reverse electrodialysis.
- 447 abstracts contained "electrolysis".
- 256 abstracts contained "desalination".
- 225 abstracts contained "redox flow batter" or "vanadium * batter", where "batter" included battery and batteries; "vanadium * batter" included but was not limited to "vanadium redox batter" and "vanadium flow batter".
- 182 abstracts contained "bipolar membrane" and "electrodialysis".
- 157 abstracts contained "donnan dialysis" or "diffusion dialysis".
- 104 abstracts contained "water treatment".
- 82 abstracts contained "reverse?electrodialysis", where included "reverse electrodialysis" and "reverse-electrodialysis".
- 52 abstracts contained "capacitive deionization".
- 43 abstracts contained "electrodeionization" or "electro-deionization".
- 31 abstracts contained "water purification".
- 25 abstracts contained "electro-electrodialysis".

It should be pointed that this method had its limitations. In other words, it might omit some applications. Or the current applications listed in Table 6 might have less or more number of publications than listed. That is why their meanings were introduced here.

1.8.3. Properties of ion exchange membranes

Similarly, the results in Table 7 were based on the abstracts analysis which was done using the Filter tool in Excel. The following *"?"* was used to represent any single character. The numbers had the following meanings.

- 4008 abstracts contained "properties".
- 4952 abstracts contained "conductivity".

- 3301 abstracts contained “stability”.
- 2175 abstracts contained “water uptake” or “water content”.
- 2158 abstracts contained “resistance”.
- 1600 abstracts contained “ion?exchange?capacity” or “IEC”.
- 1465 abstracts contained “permeability”.
- 1150 abstracts contained “morphology”.
- 1145 abstracts contained “thermal stability” or “thermally stable”. 999 abstracts contained “thermal stability”.
- 1033 abstracts contained “swelling”.
- 979 abstracts contained “hydrophilic”.
- 874 abstracts contained “hydrophobic”.
- 833 abstracts contained “mechanical properties”.
- 519 abstracts contained “chemical properties”.
- 466 abstracts contained “chemical stability”.
- 337 abstracts contained “oxidative stability” or “oxidation stability”. 310 abstracts contained “oxidative stability”.
- 306 abstracts contained “dimensional stability”.
- 298 abstracts contained “contact angle”.

Similarly to 1.8.2, it should be pointed that this method had its limitations. In other words, it might omit some properties. Or the current properties listed in Table 7 might have less or more number of publications than listed. That is why their meanings were introduced here.

1.8.4. Polymers for ion exchange membranes

The number listed in Table S8 was strictly limited to the corresponding polymer name itself. That is why two actually same polymers were listed simultaneously. In other words, the polymers listed in Table S8 were not necessarily mutually exclusive. Limitations: poly(ether ether ketone) could have other forms such as poly (ether ether ketone), polyether ether ketone. Similarly, polybenzimidazole could have other forms such as poly(benzimidazole), poly(2,5-benzimidazole). That is why they were not listed as a whole. In other words, it was very difficult to list the various forms of the polymers.

1.9. Keywords (DE) processing

Out of the 18166 articles, 14054 articles contained keywords in the right column (i.e., DE column in Excel). As is known, an article could have several keywords. These keywords were originally contained in one cell for one article. Therefore, similarly to the C1 column, the DE column was separated into several columns so that one keyword was contained in one cell. Again, this was realized through the Text To Columns tool in Excel, using semicolon as the delimiter. For example, before processing, the cell content of an article sample was read as follows:

PEM fuel cells;Bipolar plates;Flow channel design

After processing, the cell content of this article sample was separated into three cells, which were read as follows:

In one cell:

PEM fuel cells

In another cell:

Bipolar plates

In the third cell:

Flow channel design

The next step was, all the keywords in different columns were put into one column. Then, the Pivot Table tool in Excel was used to determine the count of each keyword. There were 22674 different keywords which appeared 68992 times in total in the 14054 articles. The RANK function in Excel was used to calculate the rank of each keyword in terms of articles number. Alternatively, BibExcel can also be used to get the above results (frequency distribution in the .cit file).

1.10. Research Areas (SC) processing

The procedures for SC were similar to the procedures for DE.

2. Data Processing Results (Supplementary)

Table S2: Number of countries/regions in terms of number of articles published

Number of Articles	Number of Countries/Regions
no less than 20	52
no less than 50	37
no less than 100	30
no less than 150	21
no less than 200	20
no less than 250	15
no less than 300	14
no less than 400	13
no less than 500	10
no less than 1000	5
no less than 2000	2

Table S3: Number of countries/regions in terms of percentage of articles published

Number of Articles	Number of Countries/Regions
no less than 0.10%	52
no less than 0.50%	31
no less than 1.00%	20
no less than 1.50%	14
no less than 2.00%	13
no less than 2.50%	13
no less than 3.00%	8
no less than 4.00%	8
no less than 5.00%	6
no less than 10.00%	2
no less than 20.00%	1

Table S4: The top 10 most collaborating country pairs

Country 1	Country 2	No. of Collaborations
China	USA	291
South Korea	USA	145
Canada	China	135
Canada	USA	113
France	Russia	80
China	Japan	77
China	UK	77
Japan	USA	72
China	Singapore	67
China	South Korea	66

Table S5: Number of research institutes in terms of number of articles published

Number of Articles	Number of Research Institutes
no less than 20	324
no less than 50	115
no less than 100	34
no less than 150	15
no less than 200	5
no less than 250	2
no less than 300	1

Table S6: Number of research institutes in terms of percentage of articles published

Number of Articles	Number of Research Institutes
no less than 0.10%	370
no less than 0.25%	128
no less than 0.50%	42
no less than 0.75%	20
no less than 1.00%	9
no less than 1.25%	3
no less than 1.50%	1

Table S7: The top 10 most collaborating research institute pairs

Institute 1	Institute 2	No. of Collaborations
Chinese Acad Sci	Univ Chinese Acad Sci	65
Oak Ridge Natl Lab	Univ Tennessee	37
Collaborat Innovat Ctr Chem Sci & Engn Tianjin	Tianjin Univ	37
Wageningen Univ	Wetsus	36
CNRS	Univ Grenoble Alpes	33
Korea Inst Sci & Technol	Korea Univ	32
Korea Inst Sci & Technol	Seoul Natl Univ	32
Arak Univ	Razi Univ	31
Chinese Acad Sci	Jilin Univ	31
Natl Res Council Canada	Simon Fraser Univ	29

Table S8: Common studied polymers for ion exchange membranes

Polymer	Number of abstracts
Polybenzimidazole	408
Poly(ether ether ketone)	364
Polystyrene	341
Polyimide	304
Poly(arylene ether sulfone)	280
Polysulfone	256
Poly(vinyl alcohol)	224
Polyethylene	218
Polytetrafluoroethylene	174
Polyaniline	153
Poly(vinylidene fluoride)	128
Poly(ether sulfone)	111
Polyvinyl alcohol	103
Poly(arylene ether)	99
Poly(2,6-dimethyl-1,4-phenylene oxide)	86
Polypyrrole	85
Poly(arylene ether ketone)	83
PEEK	603
SPEEK	506
PBI	502
PVA	367
PTFE	331
PVDF	314

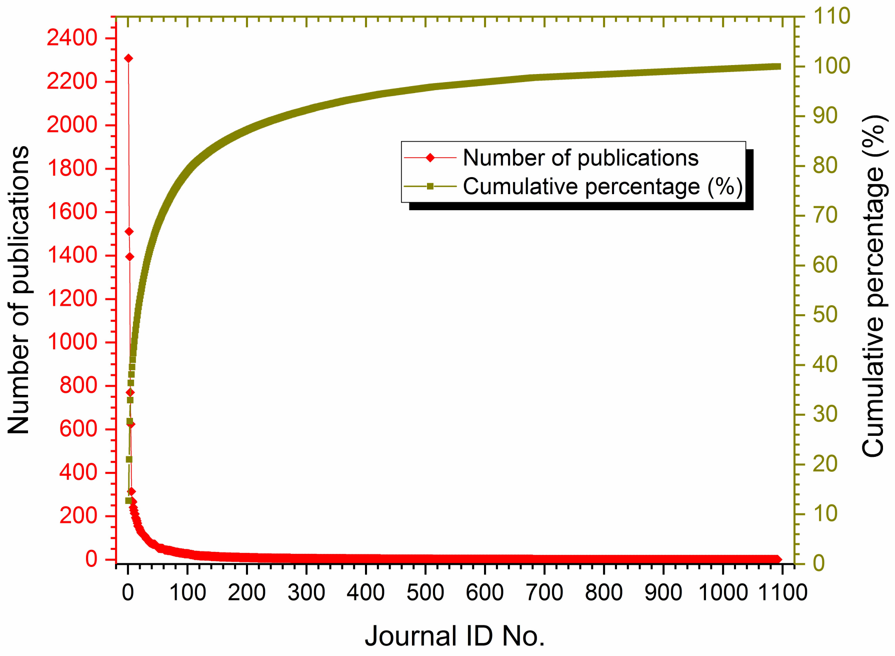

Figure S1: Number of publications by different journals. The journal ID No. was also the ranking of these journals in terms of publications, from the highest being No. 1 to the lowest being No. 1092. If two journals had the same number of publications, then they were ranked in alphabetical order.

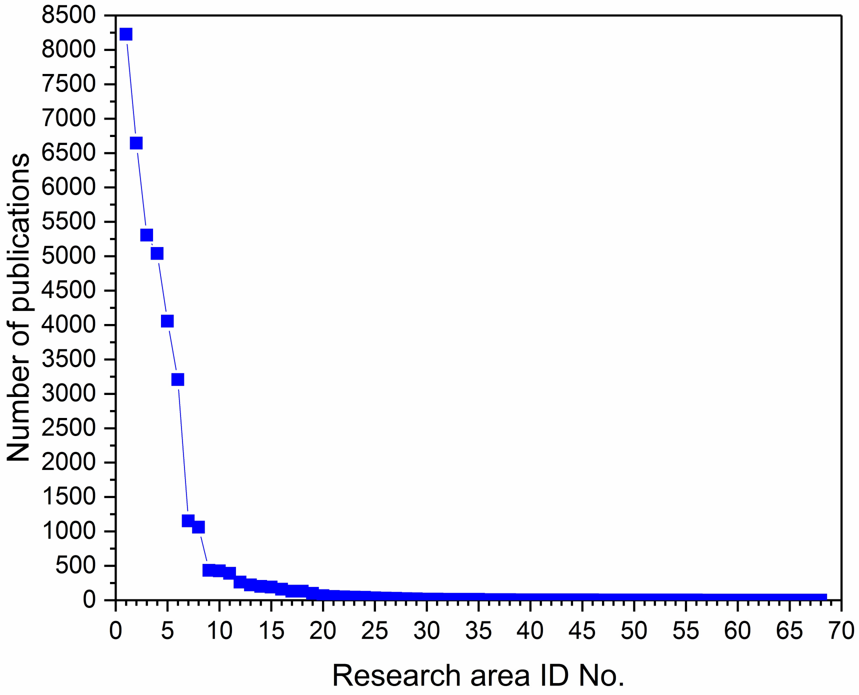

Figure S2: Number of publications among different research areas. The research area ID No. was also the ranking of these research areas in terms of publications, from the highest being No. 1 to the lowest being No. 68. If two research areas had the same number of publications, then they were ranked in alphabetical order.
